# Modeling the Influence of Mites on Honey Bee Populations

**DOI:** 10.3390/vetsci7030139

**Published:** 2020-09-21

**Authors:** David J. Torres, Nicholas A. Torres

**Affiliations:** 1 Department of Mathematics and Physical Science, Northern New Mexico College, Española, NM 87532, USA; 2 Walker Department of Mechanical Engineering, The University of Texas at Austin, Austin, TX 78712, USA; natorres@utexas.edu

**Keywords:** honey bee model, *Varroa destructor*, grooming, drones

## Abstract

The *Varroa destructor* mite has been associated with the recent decline in honey bee populations. While experimental data are crucial in understanding declines, insights can be gained from models of honey bee populations. We add the influence of the *V. destructor* mite to our existing honey bee model in order to better understand the impact of mites on honey bee colonies. Our model is based on differential equations which track the number of bees in each day in the life of the bee and accounts for differences in the survival rates of different bee castes. The model shows that colony survival is sensitive to the hive grooming rate and reproductive rate of mites, which is enhanced in drone capped cells.

## 1. Introduction

The decline of honey bees (*Apis mellifera*) has been associated with many factors, which include the *Varroa destructor* and *Varroa jacobsoni* mite and the viruses that they transmit, the microsporidia fungus *Nosema ceranae*, pesticides, including neonicotinoids, and bee management practices [1,2,3]. Here, we study the effect of the *V. destructor* mite with a mathematical model. The life cycle of the *V. destructor* mite is composed of a phoretic phase and a reproductive phase. In the phoretic phase, mites remain attached to adult bees where they feed on adult bee fat body tissue [4]. In the reproductive phase, the foundress female mite enters a worker brood or drone cell just before it is capped and lays eggs. Mites increase the mortality rate of bees, especially if they transmit a virus (e.g., the deformed wing virus and the acute paralysis virus) [5].

Models of honey bees have been developed by several authors [6,7,8,9,10,11,12]. Other authors have developed models to describe the growth of mites within honey bee colonies. Calis et al. [13] extends the model of Fries et al. [14] to study the growth of *V. jacobsoni* in colonies. Mites use available brood cells for reproduction but do not affect the bee population. Mite invasion rates are based on data from Boot et al. [15]. The model accounts for differences in mite mortality in the summer and winter as well as mite fertility in drone and worker cells. Wilkinson and Smith [16] use difference equations for adult bees and phoretic mites to study the growth rate of mites and perform sensitivity studies to study the effect of seasonal variation, the amount of drone brood, and post-capping times. Their model bases the growth of adult bees on the egg laying rate of the queen and similar to Calis et al. [13] accounts for differences in the invasion and reproductive rates of mites in worker and drone brood.

Many honey bee models include the effects of the *V. destructor* mite and the viruses that they transmit on the honey bee colony. DeGrandi-Hoffman and Curry [17] create a validated mathematical model that includes the effects of miticides. Martin [18] constructs a model of viral infection which uses mites as vectors. The model modifies the model developed by DeGrandi-Hoffman [8] and integrates meteorological conditions. Survival rates used are based on data from Fukuda and Sakagami [19]. Sumpter and Martin [20] create a model based on differential equations which tracks healthy hive bees, hive bees which acquire a virus, mites, and virus carrying mites. Hive bees are defined to be the adult worker bees that live inside the hive. Kang et al. [21] propose a model that includes parasitism, virus transmission terms, and allee effects. Ratti et al. [22] assemble a model of four differential equations, which tracks healthy and virus infested mites, and healthy and virus infested worker bees. Dénes and Ibrahim [23] use differential equations to model forager bees and three compartments of hive bees (susceptible, infested with virus free mites, and infested with virus-infected mites).

Torres et al. [24] develop a model based on differential equations that tracks each day in the life of the bee and uses different survival rates for each of the different bee castes. Th survival rates are affected by the relative number of bees in each caste. For example, the brood survival rate is decreased if there is insufficient hive bees. The model is unique in that it accounts for the brood and ethyl oleate pheromone by slowing or accelerating the maturation of hive bees into foraging bees. In this article, we add the *V. destructor* species to our existing model. This modification entails adding differential equations for the mite population, the drone population, and classes of infested pupae, drones, hive, and forager bees.

## 2. Mathematical Model

We begin with the fundamental Equation (1) from our transient model [24]

(1) $$\frac{dB_{i}}{dt}=\left(S_{i-1}B_{i-1}-B_{i}\right)a_{i},\;\;\;1\leq i\leq 55$$

where *i* refers to the age of the bee in days, $S_{i}$ is the daily survival rate of a bee that is *i* days old, and $B_{i}$ is the number of bees that are *i* days old. The term $a_{i}$ accelerates or decelerates the maturation of hive bees into foraging bees due to the presence of pheromones. Equation (1) is solved using a forward Euler difference equation,

(2) $$B_{i}^{n+1}=B_{i}^{n}+△tS_{i-1}^{n}B_{i-1}^{n}a_{i}^{n}-△tB_{i}^{n}a_{i}^{n}$$

where the subscript *n* refers to the time n△t and △t is the time step. While there are more accurate schemes, (2) benefits from simple conservation properties. The term $-△tB_{i}^{n}$ accounts for the loss of $△tB_{i}^{n}$ bees from the number of bees that are *i* days old due to natural aging in time △t. Similarly, the term $△tS_{i-1}^{n}B_{i-1}^{n}$ accounts for the movement of $△tB_{i-1}^{n}$ bees that are i−1 days old into the number of bees that are *i* days old. However, the survival factor $S_{i-1}^{n}$ reduces the number of bees $△tB_{i-1}^{n}$ that become *i* days old.

Table 1 shows how an entire bee lifespan is divided into castes based on Schmickl et al. [12] and defines the total number of days a bee spends as an egg $E_{t}\left(i\right)$, brood or larvae $L_{t}\left(i\right)$, pupae $P_{t}\left(i\right)$, hive bee $H_{t}\left(i\right)$, and foraging bee $F_{t}\left(i\right)$. Each different caste has a different associated survival rate $S_{i}$.

We also add the castes for drones $B_{i}^{D}$, as shown in Table 2. The days spent in each drone caste are reported by DeGrandi-Hoffman and Curry [17] and used in the table.

Table 3 shows the daily mortality (*m*) and survival rates (S=1−m) based on data from Schmickl et al. [12] who base their rates on experimental data from Sakagami and Fukuda [25]. Hive bees also have a higher survival rate in the winter months, which we assume to be 0.9947 using the winter mortality rate that was provided by Sumpter and Martin [20]. If there are insufficient hive bees to tend the larvae, the survival rate of the larvae $S_{i}^{reduce},\;\;4\leq i\leq 8$ is reduced according to the equation

(3) $$S_{i}^{reduce}=S_{i}r^{\alpha },\;\;r=\frac{R_{L}^{H}}{{\left(R_{L}^{H}\right)}_{ideal}},\;\mathrm{if}\;\;R_{L}^{H}<{\left(R_{L}^{H}\right)}_{ideal},\;\;4\leq i\leq 8,$$

where $S_{i}=0.99$ is the normal survival rate of larvae provided in Table 3, $R_{L}^{H}$ is the ratio of hive bees to larvae

(4) $$R_{L}^{H}=\frac{H_{t}\left(i\right)}{L_{t}\left(i\right)}=\frac{\sum_{i=21}^{i=41}B_{i}}{\sum_{i=4}^{i=8}B_{i}},$$

and ${\left(R_{L}^{H}\right)}_{ideal}$ is the ideal ratio, which is assumed to be 2 [12]. The parameter $\alpha =\frac{1}{5}$ prevents the survival rate from declining too steeply if *r* is less than but close to 1. Martin [18] also modifies the survival rate of egg, larva, and pupae, but bases the change in survival on the total number of hive bees.

The summer season is determined by the beginning $S_{begin}$ and end days $S_{end}$. In the summer, hive bees mature into foraging bees, while in the winter they remain hive bees and survive at a higher daily survival rate of 0.9947. The daily egg laying rate is determined by the parameters $S_{b}<S_{egg}<S_{begin}<S_{peak}<S_{end}<S_{e}$. We found that the equation

(5) $$L=L_{max}*\left\{\begin{array}{cc} sin^{2}\left[\frac{\pi }{2}\frac{\left(t-S_{b}\right)}{\left(S_{peak}-S_{b}\right)}\right] & \;\mathrm{if}\;S_{egg}\leq t\leq S_{peak}\\ cos^{2}\left[\frac{\pi }{2}\frac{\left(t-S_{peak}\right)}{\left(S_{e}-S_{peak}\right)}\right] & \;\;\mathrm{if}\;S_{peak}<t\leq S_{end} \end{array}\right.$$

approximately matches the growth rate of the egg laying curve of Schmickl and Crailsheim [12] and can be used with adjustable beginning and ending summer dates. The parameters $S_{b}$, $S_{egg}$, and $S_{e}$ allow the queen bee to begin and end the egg laying rate at a non-zero value in early spring and late summer. $S_{peak}$ is the day when the egg laying rate peaks for the summer. $L_{max}$ represents the maximum daily egg laying rate during the summer season.

### 2.1. Adding Mites to the Mathematical Model

In order to account for mites, we add equations to model the aging and survival of mites within the colony and assume mites survive an average of 27 days [21] in the summer when larvae brood is present

(6) $$\frac{dM_{i}}{dt}=\left(S_{i-1}^{M}M_{i-1}-M_{i}\right)-D_{i},\;\;\;1\leq i\leq 27.$$

Here, $M_{i}$ represents the number of mites that are *i* days old, and $S_{i-1}^{M}$ refers to the daily survival rate of mites. The survival rate of the mites is increased during the winter months. Martin provides the daily mortality rates of mites [26], which are 0.006 (corresponding to a survival rate of $S^{M}=0.994$) in the summer and 0.002 (corresponding to a survival rate of 0.998) in the winter.

Phoretic mites are assumed to be equally distributed among the infested hive and drone bees. We assume that, if a bee is groomed, the mites on the bee die. Mites also die in a capped cell if the pupae dies within the cell. These reductions in the mite population are incorporated in the term $D_{i}$ in (6). The product of the number of infested hive and drone bees times a maximum mite per bee value ξ determines the maximum number of phoretic mites that can be sustained by the colony.

### 2.2. Adding Drones and Infected Bee Populations to the Mathematical Model

An infested (denoted by an asterisk *) hive or forager bee is defined to be a bee with attached mites. In the case of pupae, an infested pupae harbors mites within its capped cell. We add additional equations for pupae infested with mites $B_{i}^{*},\;9\leq i\leq 20$, infested foraging bees $B_{i}^{*},\;42\leq i\leq 55$ (7), infested drone pupae $B_{i}^{D*},\;11\leq i\leq 24\;$ (8), and noninfested or “healthy” drone larvae and pupae $B_{i}^{D}$, 4≤i≤10, 11≤i≤24 (9),

(7) $$\begin{array}{ccc} \mathrm{Infested}\;\mathrm{pupae}\;\mathrm{and}\;\mathrm{foragers}: & \;\frac{dB_{i}^{*}}{dt}=S_{i-1}^{*}B_{i-1}^{*}-B_{i}^{*}, & 9\leq i\leq 20,\;\;42\leq i\leq 55, \end{array}$$

(8) $$\begin{array}{ccc} \mathrm{Infested}\;\mathrm{drone}\;\mathrm{pupae}: & \;\frac{dB_{i}^{D*}}{dt}=S_{i-1}^{*}B_{i-1}^{D*}-B_{i}^{D*}, & 11\leq i\leq 24, \end{array}$$

(9) $$\begin{array}{ccc} \mathrm{Healthy}\;\mathrm{drone}\;\mathrm{larvae}\;\mathrm{and}\;\mathrm{pupae}: & \;\frac{dB_{i}^{D}}{dt}=S_{i-1}B_{i-1}^{D}-B_{i}^{D}, & 4\leq i\leq 10,\;11\leq i\leq 24. \end{array}$$

For the healthy drone castes, we assume the same survival rates as their hive bee counterparts (see Table 3). However, we use different infested survival rates $S_{i}^{*}=1-m_{i}^{*}$ based on an increased mortality rate $m_{i}^{*}$ for the infested castes.

The equations pertaining to noninfested or healthy $B_{i}$ bees (10) and infested hive bees $B_{i}^{*}$ (11), 21≤i≤41, and healthy $B_{i}^{D}$ (12), and infested drone bees $B_{i}^{D*}$ (13), 25≤i≤45, require additional terms to account for the movement of healthy bees $B_{i}$, $B_{i}^{D}$ into the subpopulation of infested bees $B_{i}^{*}$, $B_{i}^{D^{*}}$ using the transmission rate β and the migration of infested bees back to the healthy subpopulation using the grooming rate γ

(10) $$\begin{array}{ccc} \mathrm{Healthy}\;\mathrm{hive}\;\mathrm{bees}: & \;\frac{dB_{i}}{dt}=\left(S_{i-1}B_{i-1}-B_{i}\right)a_{i}-\beta R\left(t\right)B_{i}+\gamma B_{i}^{*}, & 21\leq i\leq 41, \end{array}$$

(11) $$\begin{array}{ccc} \mathrm{Infested}\;\mathrm{hive}\;\mathrm{bees}: & \;\frac{dB_{i}^{*}}{dt}=\left(S_{i-1}^{*}B_{i-1}^{*}-B_{i}^{*}\right)a_{i}+\beta R\left(t\right)B_{i}-\gamma B_{i}^{*}, & 21\leq i\leq 41, \end{array}$$

(12) $$\begin{array}{ccc} \mathrm{Healthy}\;\mathrm{drones}: & \;\frac{dB_{i}^{D}}{dt}=S_{i-1}B_{i-1}^{D}-B_{i}^{D}-\beta R\left(t\right)B_{i}^{D}+\gamma B_{i}^{D*}, & 25\leq i\leq 45, \end{array}$$

(13) $$\begin{array}{ccc} \mathrm{Infested}\;\mathrm{drones}: & \;\frac{dB_{i}^{D*}}{dt}=S_{i-1}^{*}B_{i-1}^{D*}-B_{i}^{D*}+\beta R\left(t\right)B_{i}^{D}-\gamma B_{i}^{D*}, & 25\leq i\leq 45, \end{array}$$

where

$$R\left(t\right)=\frac{H_{t}^{*}\left(t\right)}{H_{t}\left(i\right)}$$

is the ratio of the number of infested hive bees to the total number of hive bees. The term R is consistent with the SIR (Susceptible, Infected, Recovered) model of infectious disease [27]. These equations allow for a hive or drone bee to be infested with mites either through infested pupae maturation or as adults through the transmission term.

Because we assume that infested hive bees are less effective in tending to larvae, we modify the ideal hive to larvae ratio ${\left(R_{L}^{H}\right)}_{ideal}$ in (3) using the weighted mean

(14) $${\left(R_{L}^{H}\right)}_{ideal}^{mod}=\frac{\left[H_{t}\left(i\right)-H_{t}^{*}\left(t\right)\right]{\left(R_{L}^{H}\right)}_{ideal}+H_{t}^{*}\left(i\right){\left(R_{L}^{H}\right)}_{ideal}^{*}}{H_{t}\left(i\right)}$$

in the presence of infested hive bees, where ${\left(R_{L}^{H}\right)}_{ideal}^{*}=3.0$.

### 2.3. Proportion of Drone Eggs

The proportion *Z* of eggs that are selected to be drone eggs is determined by the equation that was provided by Martin [18]

(15) $$Z=0.2263\;log_{10}\left(0.1D_{L}\right)\;log_{10}\left[0.006\left(H_{t}\left(t\right)+H_{t}^{*}\left(t\right)\right)\right]$$

where $D_{L}$ is the day length which is determined by the day of the year and the latitude. We find that the equation produces fairly similar rates when compared to the rates of drone egg production used by Sumpter and Martin [20] in the spring 1%, summer 3.3%, and autumn 1%. The day length is computed using the equations that were provided by the CBM model in Forsythe et al. [28] and Brock [29].

### 2.4. Mite Reproduction

While the female foundress mite lays up to 5–6 eggs within the capped cell, surviving daughter mite offspring are produced at the rate of 1.3–1.45 for worker brood and 2.2–2.6 for drone brood [5]. The reproductive rate is higher in drone brood because drone brood remain capped for a longer period [5]. Martin [26] uses 1.01 and 2.91 for the reproductive rate for mites emerging from hive worker brood and drone brood, respectively, to account for a multitude of factors including mite infertility. DeGrandi-Hoffman et al. [30] use 1.5 and 2.6 for the number of surviving daughter mite offspring in worker and drone brood and we adopt these values in our model.

Sumpter and Martin [20] assume the percentage of time mites spend in capped pupae cells is 75%. While Kang et al. [21] state that the phoretic period may last 4.5–11 days when larvae are present, Martin notes that the mean number of days a mite spends as a phoretic mite is 4–6 [26], which slightly underestimates the 75% percentage if the mite lifespan is 27 days. Our model distributes 75% of total mites to available larvae cells on the day before they are capped. Let us refer to the mites that need to be distributed to available larvae as invading mites. Invading mites remain in capped cells for 12 days in worker pupae cells and 14 days in drone pupae cells. We assume that 85% of invading mites invade drone brood first up to four mites per cell due to the high propensity (5.5 to 12.1 times) of mites to invade drone cells compared to worker cells [14]. If there are insufficient drone brood to hold the invading drone mites, the remaining mites that did not invade a drone cell and 15% of the initial pool of invading mites are then distributed to available worker brood up to four mites per cell. The reduction in mite offspring due to multiple infestations of foundress mites is determined by using the values provided by Martin [26]. Specifically the number of offspring is reduced by 0.91, 0.86, and 0.60 in cells with two, three, and four foundress mites per cell in drone cells and 0.84, 0.65, and 0.66 in cells with two, three, and four foundress mites per cells in worker cells. The survival rates of the worker and drone pupae with multiple mite infestations are also reduced according to DeGrandi-Hoffman and Curry [17]. The survival rates are reduced 10%, 20%, and 40% for pupae with two, three, or four mites per cell respectively. A female mite usually has 2–3 reproductive cycles during her lifetime [5]. Martin [26] assumes the number of reproductive cycles for a mite to be 2.4. DeGrandi-Hoffman and Curry [17] only allow 60% of mites to invade a cell a second time. We only consider mites that are 16 days old or younger to be eligible for brood invasion, which allows for a maximum of two invasions.

### 2.5. Code and Computational Times

The code is written in MATLAB but also runs with Octave. Octave can be downloaded for free at gnu.org/software/octave. The free code can be downloaded at the github link: https://github.com/davytorres/beecode-with-mites/. Computational times vary on different computers and versions of MATLAB. A two-year simulation with a time step of 0.1 days runs in approximately 0.07 s on a Dell Ultrabook with a 2.6 GHz Intel Core i5 processor with 8 GB of RAM with MATLAB R2018b, while the same simulation runs in approximately 0.12 s on a MacBook Air with a 1.6 GHz Intel Core i5 processor with 8 GB of memory with MATLAB R2017a. Computational times can increase by orders of magnitude with earlier versions of MATLAB or Octave. The same simulation runs in approximately 4 s on a Dell Inspiron 3671 with a 2.9 GHz Intel Core i5 processor with 8 GB of installed memory with MATLAB R2014a and in 83.7 s with Octave 5.2.0 on the same machine. Despite the differences in computational times, the algorithm is fairly efficient and sensitivity studies can be conducted with two interacting variables.

## 3. Results

**Figure 1:**
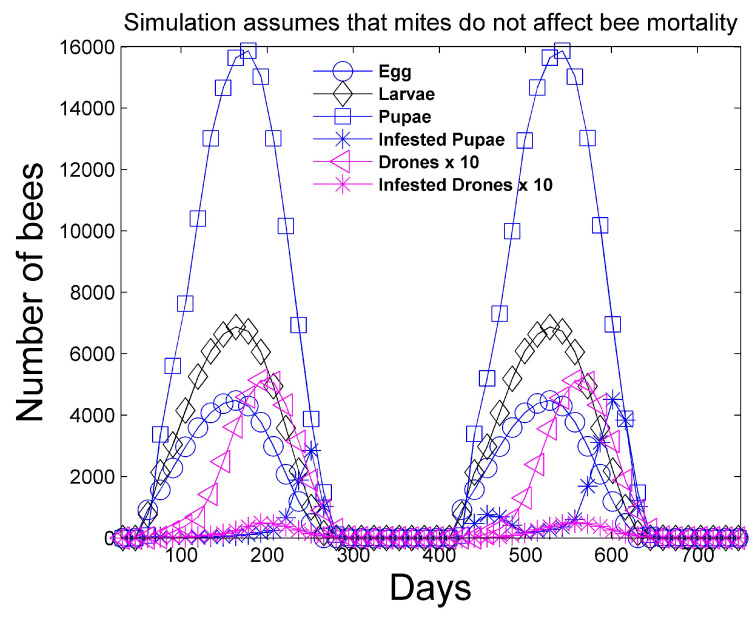
Two-year simulation of a honey bee colony with mites using the simulation parameters in Table 4 and Table 5. The number of eggs, larvae, total pupae, infested pupae, total drones, and infested drones is shown. Mites do not affect the survival rate of any bee caste in this simulation. The number of drones is multiplied by 10 for plotting purposes.

**Figure 2:**
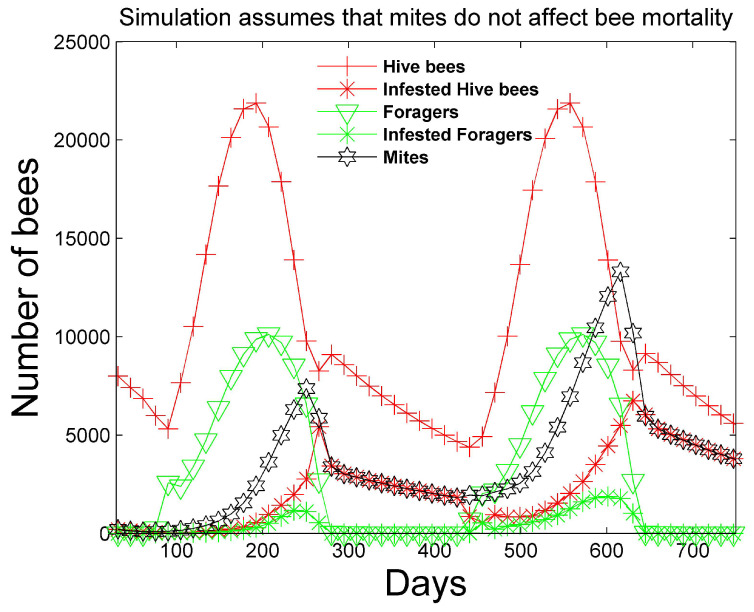
Two-year simulation of a honey bee colony with mites using the simulation parameters in Table 4 and Table 5. The number of total hive bees, infested hive bees, total foraging bees, and infested foraging bees is shown. Mites do not affect the survival rate of any bee caste in this simulation. The number of mites closely matches the number of infested hive bees after the summer season ends when one mite remains attached to each infested hive bee.

**Figure 3:**
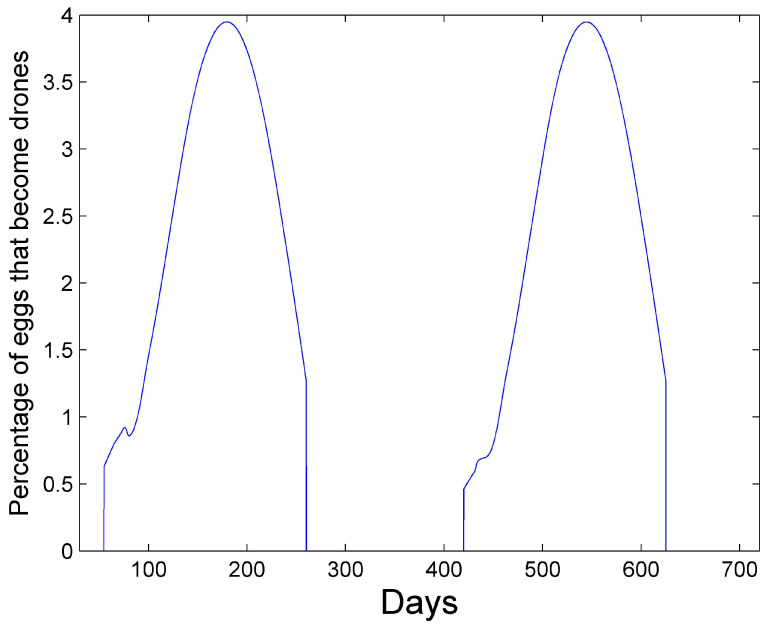
Percentage of eggs that become drone eggs using Equation (15) at latitude 35${}^{\circ }$. The average percent value over the two years is 2.5% during the summer.

**Figure 4:**
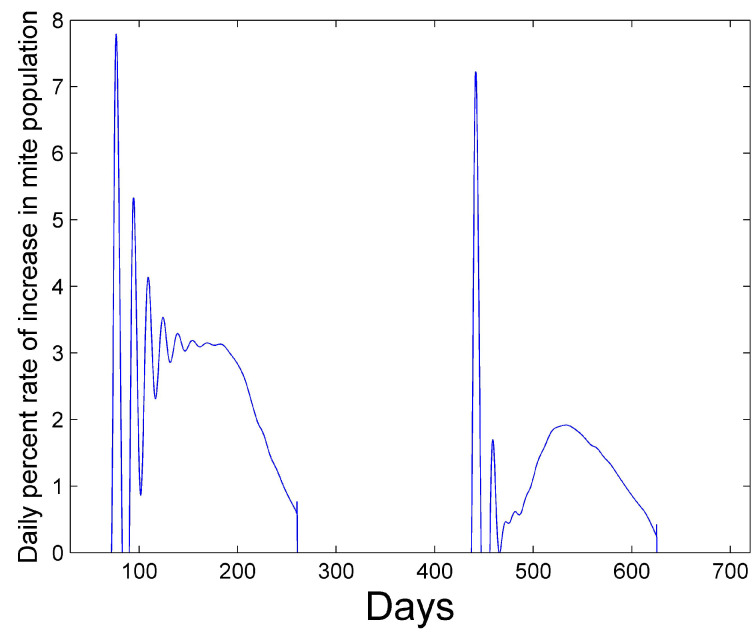
Daily percent rate of increase in mite population as defined by (16). After a period of oscillation, the rate relaxes. The average rate in the first year is 2.6% and the average rate in the second year is 1.3%.

**Figure 5:**
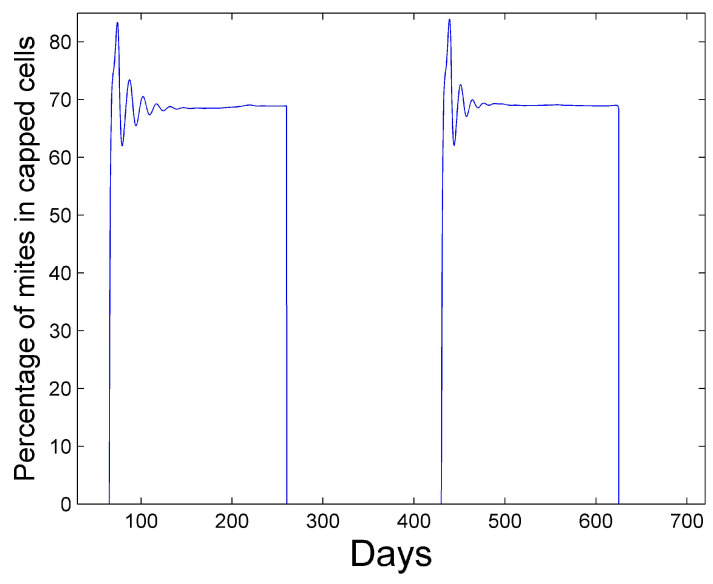
Percentage of mites in capped cells during summer season.

Figure 1 and Figure 2 show a two-year simulation of a honey bee colony with mites with separate plots for eggs $E_{t}\left(i\right)$, larvae $B_{t}\left(i\right)$, total pupae $P_{t}\left(i\right)$, infested pupae $P_{t}^{*}\left(i\right)$, total hive bees $H_{t}\left(i\right)$, infested hive bees $H_{t}^{*}\left(i\right)$, total foraging bees $F_{t}\left(i\right)$, and infested foraging bees $F_{t}^{*}\left(i\right)$ (see Table 1). Note that the total caste subpopulation (e.g., $H_{t}\left(i\right)$) includes the infected caste (e.g., $H_{t}^{*}\left(i\right)$) subpopulation. The survival rates in Table 3 and the simulation parameters in Table 4 and Table 5 are used. In the simulation, the mites do not affect the survival rates of any of the bee castes, and the hive is provided with an infinite supply of food. Differences in the number of bees in each caste and the height of each caste graph are mostly determined by the number of days bees spend in each caste, but are also determined to a lesser degree on the survival rate of each caste and pheromones in the hive caste. The number of drones $D_{t}\left(i\right)$ is also shown, but multiplied by 10 for plotting purposes, since the number of drones is relatively small. The location of the peaks are staggered beginning with the egg caste, and continuing with the larvae, pupae, hive, and foraging castes and reflect the maturation time spent in each caste. We note that the mite population peaks later than the peak of the foraging bee population. DeGrandi-Hoffman et al. [30] also show that mite populations are the largest in September through November. Figure 3 shows the percentage of eggs that become drones using (15) at latitude 35${}^{\circ }$. The graph has an average percent value of 2.5% over the two years during the summer. Figure 4 shows the positive values of the percent daily increase in the mite population,

(16) $$100*max\left\{\left[\frac{M_{t}\left(i+1\right)}{M_{t}\left(i\right)}\right]-1,0.0\right\},\;\;M_{t}\left(i\right)=\sum_{i=1}^{27}M_{i},$$

corresponding to the mite population curve in Figure 2. After a period of oscillation, the rate relaxes. The average rate in the first year is 2.6% and the average rate in the second year is 1.3%. The reported percent rate of growth of the mite population in virus-free colonies ranges from 2.1% [31,32] to 2.2–2.5% [33]. Figure 5 shows the percentage of mites that spend their time in capped cells during the summer season. We see that approximately 70% of the mites reside within capped cells. Martin [26] predicts that 50% to 70% of mites reside within sealed brood with his model.

**Figure 6:**
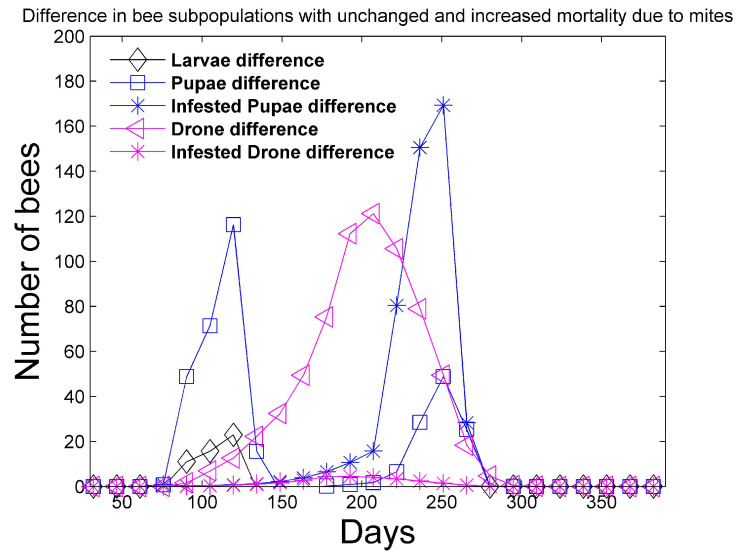
Two-year simulation of a honey bee colony with mites using the simulation parameters in Table 4 and Table 5. Mites increase the mortality rates in Table 3 of infested pupae, hive, drone, and foragers by four times. Only the first year is shown since the colony does not survive past the first year. In this figure, the **difference** in the bee subpopulations (larvae, total pupae, infested pupae, total drones, infested drones) with unaffected mortality (Figure 1 and Figure 2) and the bee subpopulations with increased mortality is shown.

**Figure 7:**
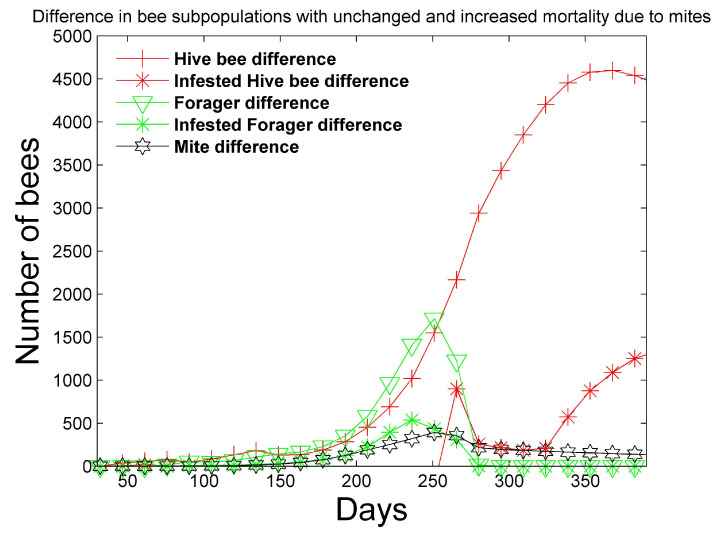
Two-year simulation of a honey bee colony with mites using the simulation parameters in Table 4 and Table 5. Mites increase the mortality rates in Table 3 of infested pupae, hive, drone, and foragers by four times. Only the first year is shown since the colony does not survive past the first year. In this figure, the difference between the number of bee subpopulations (total hive bees, infested hive bees, total foraging bees, and infested foraging bees) with unchanged mortality (Figure 1 and Figure 2) and the bee subpopulations with increased mortality is plotted.

In Figure 6 and Figure 7, a simulation is performed with mites under the same conditions as Figure 1 and Figure 2, except the mites increase the mortality rates of the infested pupae, hive, drone, and forager castes shown in Table 3 by four times. We also apply (14), where ${\left(R_{L}^{H}\right)}_{ideal}^{*}=3.0$ in order to require additional infested hive bees to effectively attend to larvae. This requirement is not enforced in Figure 1 and Figure 2, where ${\left(R_{L}^{H}\right)}_{ideal}^{*}={\left(R_{L}^{H}\right)}_{ideal}=2$. The **differences** in the bee castes are plotted. For example, in regard to hive bees, the difference

$$H_{t}\left(i\right)\left[\mathrm{with}\mathrm{unchanged}\mathrm{mortality}\right]-H_{t}\left(i\right)\left[\mathrm{with}\mathrm{increased}\mathrm{mortality}\right]$$

is plotted in Figure 7. Figure 6 shows that the simulation with unchanged mortality rates (Figure 1 and Figure 2) has larger numbers of larvae, pupae, and drones when compared to the simulation with increased bee mortality. However, the differences are less than 200 in each category. Figure 7 shows that more mites exist in the simulation with unchanged bee mortality, because the mites are negatively affected by increased bee mortality. In addition, the simulation with unchanged bee mortality has significantly larger numbers of hive and foraging bees. The number of hive bees that persist late into the summer season is significantly reduced with increased bee mortality and the reduced number of hive bees does not allow the colony to survive past the first year.

**Figure 8:**
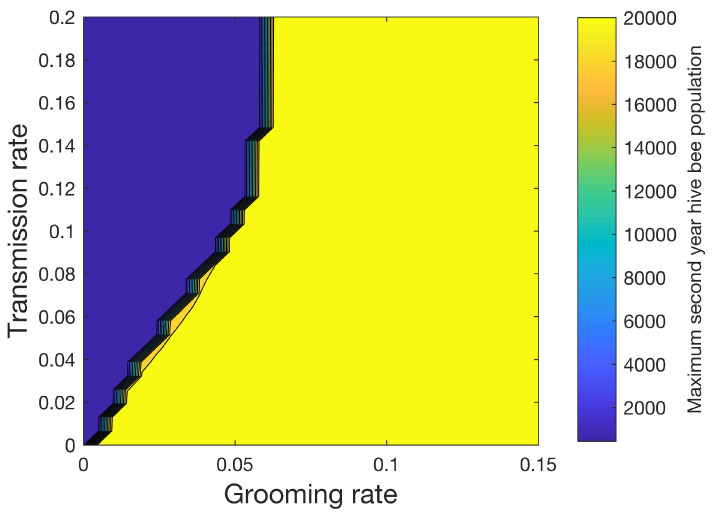
Effect of grooming rate γ and transmission rate β (10)–(13) on the second year maximum hive bee population. The transmission rate models the rate at which uninfested bees become infested. The grooming rate models the rate at which infested bees becomes uninfested. A sharp line divides the conditions under which the colony can be sustained (yellow) versus when the colony perishes (blue).

Figure 8 shows how the maximum number of hive bees varies in the second year when 32 different values of the grooming rate ranging from 0 to 0.15 and 32 different values of transmission rate ranging from 0 to 0.2 are used for a total of 1024 simulations. In this sensitivity study, mites increase the mortality rates of the infested pupae, hive, drone, and forager castes shown in Table 3 by four times. We also apply (14), where ${\left(R_{L}^{H}\right)}_{ideal}^{*}=3.0$. With the exceptions noted above, the simulation parameters in Table 3, Table 4 and Table 5 are used. A sharp line separates the conditions under which the colony can be sustained (yellow) from the conditions under which the colony perishes (blue) beyond the first year. Figure 8 shows the importance of the grooming rate in sustaining the colony.

**Figure 9:**
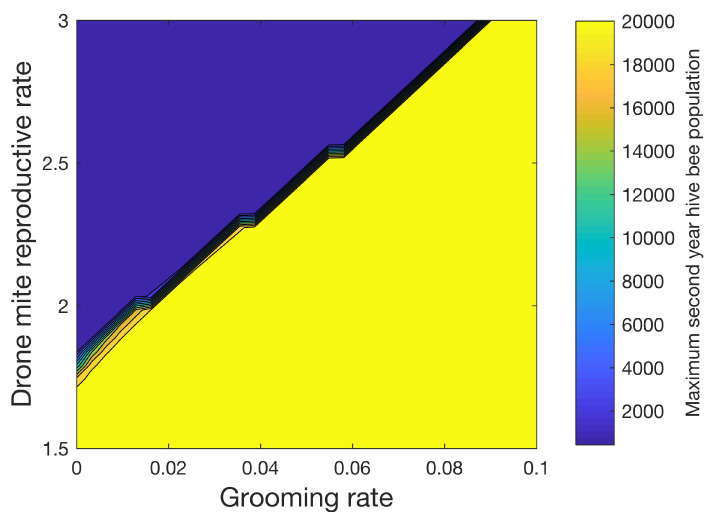
Effect of grooming rate γ (10)–(13) and drone reproductive rate on the second year maximum hive bee population. The grooming rate models the rate at which infested bees becomes uninfested. The transmission rate β=0.2. A sharp line divides the conditions under which the colony can be sustained (yellow) versus when the colony perishes (blue). The figure shows that drones play an important role in sustaining the mite population due to the higher reproductive rate of mites in drone capped cells.

Figure 9 shows how the maximum number of hive bees varies in the second year when 32 different grooming rates ranging from 0 to 0.1 and 32 different reproductive rates ranging from 1.5 to 3.0 for the drone foundress mite are used. As with Figure 8, in this sensitivity study, mites increase the mortality rates of the infested pupae, hive, drone, and forager castes by four times. A transmission rate of β=0.2 is used. We also apply (14) where ${\left(R_{L}^{H}\right)}_{ideal}^{*}=3.0$. With the exceptions noted above, the simulation parameters in Table 3, Table 4 and Table 5 are used. Again, a sharp line separates the conditions under which the colony can be sustained. Figure 9 shows the importance of the higher reproductive level of mites in drone cells. Mite populations are significantly reduced in simulations of colonies without drones, despite the relatively small number of drones when compared to hive bees. We note that beekeepers do remove drone brood in practice to control mites [34].

## 4. Discussion

We have developed a mathematical model of a honey bee colony with mites, which tracks healthy and infested pupae, hive bees, drones, and foragers. The model is based on differential equations that solve for the number of bees in each day in the life of the bee. Model simulations shed light on the effect of different parameters on colony sustainability. Specifically, the grooming rate, transmission rate, and reproductive rate of the foundress mites are all important parameters that determine whether a colony will survive or perish.

Our model shows that the mite population is significantly reduced in the absence of drones despite the relatively low number of drones compared to hive bees. The importance of drone brood to mite population growth has also been reported in other models [13,17,35]. Our model also shows that grooming is an effective mechanism for controlling the growth of mites. The absence of grooming in the model can lead to very high populations of mites and the importance of grooming to bee survival has been noted by other authors [13,36,37].

## 5. Conclusions

The observations from our mathematical model could lead to continued or new experimental investigations in order to test the impact drone reduction [23] and increased grooming rate have on colony sustainability and ultimately help guide bee management processes (e.g., removal of drone brood and selective breeding) to improve colony health.

Future research efforts would be aimed at adding the influence of viruses and the increased mortality that they bring to specific castes. A computational comparison of available models of mite growth would also be useful to quantitatively compare the differences and similarities of facets within each model.

## Figures and Tables

**Table 1:** Day ranges used to calculate bee demographics.

Sum over $B_{i}$	Bee Caste	Number of Days in Caste
$E_{t}\left(i\right)\equiv \sum_{i=1}^{i=3}B_{i}$	Egg (E)	3
$L_{t}\left(i\right)\equiv \sum_{i=4}^{i=8}B_{i}$	Brood or larvae (L)	5
$P_{t}\left(i\right)\equiv \sum_{i=9}^{i=20}B_{i}$	Pupae (P)	12
$H_{t}\left(i\right)\equiv \sum_{i=21}^{i=41}B_{i}$	Hive (H)	21
$F_{t}\left(i\right)\equiv \sum_{i=42}^{i=55}B_{i}$	Forager (F)	14

**Table 2:** Day ranges used to calculate drone demographics.

Sum over $B_{i}^{D}$	Bee Caste	Number of Days in Caste
$E_{t}^{D}\left(i\right)\equiv \sum_{i=1}^{i=3}B_{i}^{D}$	Drone Egg $\left(E^{D}\right)$	3
$L_{t}^{D}\left(i\right)\equiv \sum_{i=4}^{i=10}B_{i}^{D}$	Drone Larvae $\left(L^{D}\right)$	7
$P_{t}^{D}\left(i\right)\equiv \sum_{i=11}^{i=24}B_{i}^{D}$	Drone Pupae $\left(P^{D}\right)$	14
$D_{t}\left(i\right)\equiv \sum_{i=25}^{i=45}B_{i}^{D}$	Adult Drone (D)	21

**Table 3:** Daily mortality (*m*) and survival (*S* = 1 − *m*) rates.

$m_{\mathrm{egg}}$	$m_{\mathrm{larvae}}$	$m_{\mathrm{pupae}}$	$m_{\mathrm{hive}}$	$m_{\mathrm{forager}}$
0.03	0.01	0.001	0.015	0.045
$S_{\mathrm{egg}}$	$S_{\mathrm{larvae}}$	$S_{\mathrm{pupae}}$	$S_{\mathrm{hive}}$	$S_{\mathrm{forager}}$
0.97	0.99	0.999	0.985	0.955

**Table 4:** Simulation parameters.

Initial number of hive bees	8000
Simulation start date	1 February
Maximum egg laying rate	1600
Ideal hive bee to brood ratio	2
Ideal hive bee to forager ratio	2.3
Hive bee daily survival rate in winter	0.9947
Latitude	35${}^{\circ }$
Time step △t	0.1 day
Length of summer	6 March to 17 September
$S_{b}$, $S_{egg}$, $S_{peak}$, $S_{e}$	16, 55, 162, 272 days

**Table 5:** Simulation parameters with mites.

Initial number of mites	200
Lifetime of mite	27 days
Mite daily survival rate in summer	0.994
Mite daily survival rate in winter	0.998
Reproductive rate of foundress mite in drone brood	2.6
Reproductive rate of foundress mite in worker brood	1.5
Maximum number of mites in capped brood	4
Maximum number of mites per infested hive or drone bee ξ	6
Grooming rate γ	0.05
Transmission rate β	0.25

## References

[1] Evans J.D., Spivak M. (2010). Socialized medicine: Individual and communal disease barriers in honey bees. J. Invertebr. Pathol..

[2] Johnson R.M. (2015). Honey bee toxicology. Annu. Rev. Entomol..

[3] van Engelsdorp D., Evans J.D., Saegerman C., Mullin C., Haubruge E., Nguyen B.K., Frazier M., Frazier J., Cox-Foster D., Chen Y. (2009). Colony Collapse Disorder: A Descriptive Study. PLoS ONE.

[4] Ramsey S.D., Ochoa R., Bauchan G., Gulbronson C., Mowery J.D., Cohen A., Lim D., Joklik J., Cicero J.M., Ellis J.D. (2019). *Varroa destructor* feeds primarily on honey bee fat body tissue and not hemolymph. Proc. Natl. Acad. Sci. USA.

[5] Rosenkranz P., Aumeier P., Ziegelmann B. (2010). Biology and control of *Varroa destructor*. J. Invertebr. Pathol..

[6] Becher M.A., Osborne J.L., Thorbek P., Kennedy P.J., Grimm V. (2013). Towards a systems approach for understanding honeybee decline: A stocktaking and synthesis of existing models. J. Appl. Ecol..

[7] Becher M.A., Grimm V., Thorbek P., Horn J., Kennedy P.J., Osborne J.L. (2014). BEEHAVE: A systems model of honeybee colony dynamics and foraging to explore multifactorial causes of colony failure. J. Appl. Ecol..

[8] DeGrandi-Hoffman G., Roth S.A., Loper G.L., Erickson E.H. (1989). BEEPOP: A honeybee population dynamics simulation model. Ecol. Model..

[9] Khoury D.S., Myerscough M.R., Barron A.B. (2011). A Quantitative Model of Honey Bee Colony Population Dynamics. PLoS ONE.

[10] Khoury D.S., Barron A.B., Myerscough M.R. (2013). Modelling food and population dynamics in honey bee colonies. PLoS ONE.

[11] Russell S., Barron A.B., Harris D. (2013). Dynamic modelling of honey bee (*Apis mellifera*) colony growth and failure. Ecol. Model..

[12] Schmickl T., Crailsheim K. (2007). HoPoMo: A model of honeybee intracolonial population dynamics and resource management. Ecol. Model..

[13] Calis J.N.M., Fries I., Ryrie S.C. (1999). Population modelling of *Varroa jacobsoni* Oud. Apidologie.

[14] Fries I., Camazine S., Sneyd J. (1994). Population dynamics of *Varroa jacobsoni*: A model and a review. Bee World.

[15] Boot W.J., Beetsma J., Calis J.N.M. (1994). Behaviour of *Varroa* mites invading honey bee brood cells. Exp. Appl. Acarol..

[16] Wilkinson D., Smith G.C. (2001). A model of the mite parasite, *Varroa destructor*, on honeybees (*Apis mellifera*) to investigate parameters important to mite population growth. Ecol. Model..

[17] DeGrandi-Hoffman G., Curry R.A. (2000). Mathematical Model of Varroa Mite (Varroa Destructor Anderson and Trueman) and Honeybee (*Apis Mellifera* L.) Population Dynamics. Internate J. Acarol..

[18] Martin S.J. (2001). The role of *Varroa* and viral pathogens in the collapse of honeybee colonies: A modelling approach. J. Appl. Ecol..

[19] Fukuda H., Sakagami S. (1968). Worker brood survival in honey bees. Res. Popul. Ecol..

[20] Sumpter D.J.T., Martin S.J. (2004). The dynamics of virus epidemics in Varroa-infested honey bee colonies. J. Anim. Ecol..

[21] Kang Y., Blanco K., Davis T., Wang Y., DeGrandi-Hoffman G. (2016). Disease dynamics of honeybees with Varroa destructor as parasite and virus vector. Math. Biosci..

[22] Ratti V., Kevan P.G., Eberl H.J. (2013). A mathematical model for population dynamics in honeybee colonies infested with *Varroa destructor* and the Acute Bee Paralysis Virus. Can. Appl. Math. Q..

[23] Dénes A., Ibrahim M.A. (2019). Global dynamics of a mathematical model for a honeybee colony infested by virus-carrying *Varroa* mites. J. Appl. Math. Comput..

[24] Torres D.J., Ricoy U.M., Roybal S. (2015). Modeling honey bee populations. PLoS ONE.

[25] Sakagami S.F., Fukuda H. (1968). Life tables for worker honeybees. Res. Popul. Ecol..

[26] Martin S. (1998). A population model for the ectoparasitic mite *Varroa jacobsoni* in honey bee *Apis mellifera* colonies. Ecol. Model..

[27] Kermack W.O., McKendrick A.G. (1927). A contribution to the mathematical theory of epidemics. Proc. R. Soc. Lond. A.

[28] Forsythe W.C., Rykiel E.J., Stahl R.S., Wu H., Schoolfield R.M. (1995). A model comparison for daylength as a function of latitude and day of year. Ecol. Model..

[29] Brock T.D. (1981). Calculating solar radiation for ecological studies. Ecol. Model..

[30] DeGrandi-Hoffman G., Ahumada F., Zazueta V., Chambers M., Hidalgo G., DeJong E.W. (2016). Population growth of *Varroa destructor* (Acari: Varroidae) in honey bee colonies is affected by the number of foragers with mites. Exp. Appl. Acarol..

[31] Calatayud F., Verdu M.J. (1995). Number of adult female mites *Varroa jacobsoni* Oud. on hive debris from honey bee colonies artificially infested to monitor mite population increase. Mesostigmata: Varroidae Exp. Appl. Acarol..

[32] Kraus B., Page R.E. (1995). Population growth of *Varroa jacobsoni* Oud. in Mediterranean climates of California. Apidologie.

[33] Martin S.J., Kemp D. (1997). Average number of reproductive cycles performed by *Varroa jacobsoni* in honey bee (*Apis mellifera*) colonies. J. Apic. Res..

[34] Charrière J.D., Indorf A., Bachofen B., Tschan A. (2002). The removal of capped drone brood: An effective means of reducing the infestation of varroa in honey bee colonies. Bee World.

[35] Wilkinson D., Smith G.C. (2002). Modeling the efficiency of sampling and trapping *Varroa destructor* in the drone brood of honey bees (*Apis mellifera*). Am. Bee J..

[36] Prichard D.J. (2016). Grooming by honey bees as a component of varroa resistant behavior. J. Apic. Res..

[37] Locke B. (2016). Natural *Varroa* mite-surviving *Apis mellifera* honeybee populations. Apidologie.

